# Adverse effects of COVID-19-related lockdown on pain, physical activity and psychological well-being in people with chronic pain

**DOI:** 10.1177/2049463720973703

**Published:** 2020-11-21

**Authors:** Nicholas Fallon, Christopher Brown, Hannah Twiddy, Eleanor Brian, Bernhard Frank, Turo Nurmikko, Andrej Stancak

**Affiliations:** 1 Department of Psychology, Institute of Population Health Sciences, University of Liverpool, Liverpool, UK; 2 Pain management Programme, The Walton Centre NHS Foundation Trust, Liverpool, UK; 3 Neuroscience Research Centre, The Walton Centre NHS Foundation Trust, Liverpool, UK

**Keywords:** Pain catastrophising, exercise, anxiety, depression, coronavirus, health behaviours, self-management

## Abstract

Countries across the world imposed lockdown restrictions during the COVID-19 pandemic. It has been proposed that lockdown conditions, including social and physical distancing measures, may disproportionately impact those living with chronic pain and require rapid adaptation to treatment and care strategies. Using an online methodology, we investigated how lockdown restrictions in the United Kingdom impacted individuals with chronic pain (N = 431) relative to a healthy control group (N = 88). Data were collected during the most stringent period of lockdown in the United Kingdom (mid-April to early-May 2020). In accordance with the fear-avoidance model, we hypothesised lockdown-related increases in pain and psychological distress, which would be mediated by levels of pain catastrophising. Responses indicated that people with chronic pain perceived increased pain severity, compared to their estimation of typical pain levels prior to lockdown (p < .001). They were also more adversely affected by lockdown conditions compared to pain-free individuals, demonstrating greater self-perceived increases in anxiety and depressed mood, increased loneliness and reduced levels of physical exercise (p ⩽ .001). Hierarchical regression analysis revealed that pain catastrophising was an important factor relating to the extent of self-perceived increases in pain severity during lockdown (β = .27, p < .001) and also mediated the relationship between decreased mood and pain. Perceived decreases in levels of physical exercise also related to perceptions of increased pain (β = .15, p < .001). Interestingly, levels of pain intensity (measured at two time points at pre and during lockdown) in a subgroup (N = 85) did not demonstrate a significant change. However, individuals in this subgroup still reported self-perceived pain increases during lockdown, which were also predicted by baseline levels of pain catastrophising. Overall, the findings indicate that people with chronic pain suffer adverse effects of lockdown including self-perceived increases in their pain. Remote pain management provision to target reduction of pain catastrophising and increase health behaviours including physical activity could be beneficial for this vulnerable population.

## Introduction

COVID-19 is a highly contagious disease related to the spread of SARS-CoV-2 virus.^1^ Due to the high infection and mortality rate of COVID-19, many countries implemented periods of lockdown to reduce uncontrolled spread of the virus.^2^ Lockdown of economic and social activities creates a situation of threat in vulnerable populations due to health anxiety, physical inactivity, reduced accessibility to usual care, social isolation and financial-economic uncertainty.^2,3^

It was recently proposed that the COVID-19 pandemic would substantially impact those living with chronic pain and thus require efforts to adapt treatment and care strategies.^4^ Chronic pain affects around 40% of the UK adult population^5^ and represents a significant global burden at both the individual and socioeconomic levels.^6,7^ Increased prevalence of chronic pain in the elderly and those with comorbid illness or disability^5,8^ overlaps with the highest risk for COVID-19. Empirical research is essential to capture how people living with chronic pain are affected by the current pandemic and to support efforts to develop pain management approaches in these challenging conditions, for example, online technologies to improve levels of social support, combat social isolation and offer treatment provision.^9,10^

Previous research indicates a likelihood that chronic pain populations suffer increased severity of symptoms in high-stress situations including war or the aftermath of terrorist attacks.^11,12^ If we can better understand how high-stress situations exacerbate chronic pain, we can adapt clinical strategies to mitigate the associated suffering.^13^ A likely mediator of greater pain severity resulting from high-stress situations is psychological distress,^14^ which critically impacts on the perception of pain, physical disability^15^ and overall quality of life.^16–18^ For example, anxiety augments neural processes modulating the perception of pain.^19–21^ In addition, the fear-avoidance model of chronic pain^22,23^ points to the theoretical importance of pain-related fear and catastrophising as contributors to decreased mood and physical activity, which in turn exacerbate pain symptoms. A unique characteristic of the COVID-19 lockdowns are physical and social distancing measures; these measures would be expected to impact pain symptoms via a combination of changes in physical activity levels, mood and anxiety. Reduced physical activity during the COVID-19 pandemic could exacerbate effects of psychological stress and reduce coping with anxiety and depression, especially in vulnerable populations.^24^

The impact of COVID-19 on mental health is becoming increasingly apparent. Increased psychological distress is evident in COVID-19 patients and health professionals who treat them.^25,26^ During peak lockdown conditions, sharp increases were seen in prevalence of anxiety and depression in the general adult population in China.^27^ In research from the United States and Spain, ‘stay at home’ directives and living with chronic illness are factors associated with greater risk of adverse effects.^28,29^ Considering this evidence, there is a clear need to understand how changes in psychological well-being and physical activity levels, due to the ongoing pandemic and related lockdown conditions, impact on pain experience in chronic pain populations.

## Method

### Aims and hypotheses

This study aimed to capture the effects of the COVID-19 pandemic, and corresponding UK lockdown restrictions, on pain, psychological well-being and physical activity levels in a group of participants suffering from chronic pain compared to a non-pain group. We hypothesised that lockdown conditions would cause increased levels of pain severity relative to pre-lockdown period in respondents living with chronic pain. Second, we predicted that lockdown conditions would have a greater impact on the psychological and physical well-being of people with chronic pain, relative to non-pain, respondents. Third, we hypothesised that self-perceived changes in reported pain levels could be related to levels of pain catastrophising, and changes in their psychological well-being and physical activity, in accordance with theoretical models of fear avoidance.

### Design and procedure

Participants (N = 519) took part in an online design comprising self-reporting chronic pain participants (N = 431) and a comparison sample of non-pain control participants (N = 88). The majority of participants were recruited via online advertisements. A subgroup (N = 85) of chronic pain patients were also recruited sequentially from a database of patients who had previously given the experimenters permission to be contacted for future research. This subgroup had previously proffered comparable pre-lockdown baseline data on pain and psychological measures. Responding participants were directed to the study pages which were programmed in Qualtrics software (Qualtrics, Utah, USA). First, participants read an information sheet and gave informed consent using a tick box procedure. They answered demographic questions, self-reported whether they had chronic pain and their relevant diagnosis, and answered some questions about their personal lockdown conditions such as size of household. A series of visual analogue scales (VAS) captured current pain and well-being levels before participants completed self-report differential measures indicating their self-perception of change in their pain, physical exercise and well-being relative to pre-COVID levels. Finally, participants completed a series of short, validated questionnaires to capture pain, pain-related cognition and psychological well-being (full description below). A debrief page at the end of the study provided information on the purpose of the study. They were informed of how to contact the researchers directly with any questions and we also highlighted some useful resources for those suffering pain or psychological distress during lockdown. All respondents were recruited for this study as part of an ongoing longitudinal investigation comprising six fortnightly sessions to be completed over a period of 3 months. Every participant was offered reimbursement of £3.33 for completing each session which was paid upon completion of the longitudinal data collection (maximum total £20). Payment was made in the form of a bank transfer or online shopping gift voucher depending on participant preference at the end of the longitudinal period.

### Participants and lockdown conditions

Participants (N = 519) took part. This total comprised 470 females, 45 males and 4 participants who selected ‘other’. Ages ranged from 18 to 79 years (43.98 ± 13.38, mean ± SD). Chronic pain respondents (N = 431) comprised a range of chronic pain conditions. The primary diagnosis was categorised according to the *International Classification of Diseases*, 11th Revision (ICD-11) of the World Health Organization, derived from the main cause of their pain (Table 1). Additional information was recorded when a specific diagnosis, indicated by the patient, revealed a relevant pathophysiology relating to their chronic pain. These additional details are included as ICD-11 codes in Table 1. The proportion of patients in each chronic pain category due to such diagnoses are indicated by ratios. A subgroup of chronic pain respondents (N = 85) were recruited via contacts with a local tertiary care pain clinic, having previously given agreement to be contacted for research purposes. This subgroup contributed identical online data collection as with all other participants. However, in this subgroup, baseline data on pain (10-point numerical rating scale (NRS) and psychological measures (pain catastrophising) was available for comparison. This existing data had been collected during an in-person assessment consultation to consider suitability for a pain management programme within 6 months preceding UK lockdown. Finally, a sample of age- and sex-matched non-pain control respondents (N = 88) were also collected via online advertisements.

**Table 1.** Number of patients corresponding to each diagnostic category, and disease identification code, according to ICD-11 guidelines.

Category	ICD-11 codes	No. of patients
Chronic widespread pain	MG30.01	150
Chronic primary/secondary musculoskeletal pain	MG30.02, MG 30.3, FA00.Z, FA2Z, FA8Z, FA11, FA20.Z, FA21.Z, FA34.5, FA80.Z, FB40.1, LD26.3, LD28.1Y, ME82	174 (25/149)
Chronic primary/secondary visceral pain	MG30.00, MG 30.4, DD91.0, DD95, GA10.Z	16 (2/14)
Chronic postsurgical or posttraumatic pain	MG30.2	11
Chronic neuropathic pain	MG 30.5, MG30.50, GA34.0Y, NA04.4, NA41.Z	51
Chronic primary/secondary headache or orofacial pain	MG30.03, 8A80.Z, 8A82	12
Complex regional pain syndrome	8D8A.0Z	6
Unspecified or other	MG30.Z, 4A62, 8D64.Z	11

ICD-11: *International Classification of Diseases*, 11th Revision.

All participants were based in the United Kingdom. First responses were recorded between 3.5 weeks after the initiation of UK lockdown conditions on 17 April 2020, and final responses were recorded on 12 May 2020. This period covered the most stringent level of lockdown in the United Kingdom, comprising social distancing and advice against all non-essential travel with recommendations to work from home. Exercise with social distancing was permitted once per day. Enhanced lockdown recommendations were in place for those deemed high risk.^30^ UK recommendations were relaxed on 13 May 2020 and the data collection was halted.

### Self-report measures

Participants were asked whether they currently suffered from chronic pain. Those who answered affirmatively completed follow-up questions about the intensity of their pain in the previous week using a VAS (0–100, anchors ‘No pain at all’ to ‘Extremely Severe Pain’). All participants completed further VAS scales for described levels of the following variables for the previous week: tiredness (0–100, anchors ‘Not at all’ to ‘Extremely tired’); loneliness (0–100, anchors ‘Not at all’ to ‘Extremely lonely’); and anxiety (0–100, anchors ‘Not at all’ to ‘Extremely anxious’).

Participants who reported chronic pain then completed a differential scale, where they rated perception of pain intensity in the past 7 days relative to a typical week in the pre-COVID period. Again, this utilised a VAS (0–100, anchors ‘Very much better’, centre marker ‘About the same’, to ‘Very much worse’). All participants completed differential VAS to indicate their perceived change (relative to a typical week in pre-COVID period) for the levels of mood, anxiety and exercise over the past 7 days. The items specifically asked ‘How tense, nervous or anxious have you felt? (0-100, anchors “Very much better”, centre marker “About the same”, to “Very much worse”), how depressed or blue have you felt?’ (0-100, anchors ‘Very much better’, centre marker ‘About the same’, to ‘Very much worse’), ‘how much physical exercise have you managed to take?’ (0-100, anchors ‘Very much more than usual’, centre marker ‘About the same’, to ‘Very much less than usual’). The wording for the questions and anchors for VAS differential items was adapted from similar items in the Fibromyalgia Impact Questionnaire.^31^

Participants in the chronic pain group then reported any ‘difficulties obtaining pain medication, other treatments or social care in the past two weeks’. All participants were asked whether they had experienced any illness other than chronic pain in the previous 2 weeks. They also reported whether they were self-isolating due to high-risk status, which encompassed following enhanced recommendations to completely shield oneself during lockdown.^30^

Finally, participants completed a series of brief, validated questionnaires to consider pain experience, pain cognition and psychological well-being. Specifically, these included the Pain Catastrophizing Scale (PCS),^32^ a 13-item self-report measure of negative cognitive–affective responses to anticipated or actual pain. We also delivered an adapted version of the Brief Pain Inventory (BPI) short form^33^ to assess the severity of pain and its impact on functioning. Finally, we utilised the Hospital Anxiety and Depression Scale (HADS),^34^ a commonly used 14-item self-rating scale developed to assess psychological distress with subscales for Anxiety and Depression. The adaptations to the BPI included removal of items requesting patients draw pain location, as well as items on minimal pain and medication lists. These changes were included to optimise the survey for online delivery and reduce the overall time requirement for patients.

### Ethics and data sharing

The study was conducted in line with the recommendations of the Declaration of Helsinki and was approved by the local University of Liverpool Research Ethics Committee. The data that support the findings of this study are openly available here 10.6084/m9.figshare.12424661.

## Results

### Data reduction

A total of 933 participants accessed the study. In total, 135 failed the pre-screening questions which aligned to the exclusion criteria (requiring participants to be >18 years old and resident in the United Kingdom during the pandemic). A further 21 did not provide consent after reading the information sheet. There were 65 respondents who consented to take part but did not complete a single item and a further 193 began the study but abandoned without completing a suitable amount of the items to be considered for inclusion (<90%).

### Effect of lockdown on pain intensity

Pain was measured in chronic pain respondents by evaluating their perception of average pain intensity for the past week using a 100-point VAS and also by reporting the differential on their pain intensity relative to a typical week in the pre-lockdown period. The mean pain intensity score in the chronic pain group was 66.64 ± 17.93 (mean ± SD). Univariate t-test analysis indicated that chronic pain respondents reported a statistically significant increase in their pain relative to pre-COVID period on the differential VAS (p < .001). The differential scores were numerically transformed to give a score from −100, with negative integers indicating pain decrease, to +100, with positive integers indicative of pain increase (0 values were equal to no perceived change). The mean change for the chronic pain group was 33.64 ± 37.20 (mean ± SD), indicating a significant self-perceived increase in pain compared to the period before the pandemic; t(431) = 18.79, p *<* .001.

To further investigate potential changes in pain intensity relative to pre-COVID periods, a pre-post lockdown comparison was conducted for the subgroup of 85 participants for whom baseline data was available. This group had agreed to take part having already provided previous data in the pre-COVID period (in the 6 months prior to lockdown in the United Kingdom). In this subgroup of participants, a within-subjects t-test was utilised to compare current pain intensity with previous data. For comparison with existing baseline NRS data, the current pain score was converted from the 100-point VAS to a 10-point NRS equivalent by dividing by 10 and then rounding to nearest whole integer. Five participants had some missing data for the comparison of pain scores and were omitted from the pain comparison. To consider whether changes in psychological pain constructs might also account for different perception of pain levels, we also compared pre-and-post PCS scores (N = 85). Table 2 illustrates mean pain intensity ratings and PCS scores for each time point. Results indicate that patients in the subgroup did not demonstrate a significant increase in reported pain intensity levels compared to data given during the baseline period; t(79) = −1.45, p *=* .15. Likewise, there was no significant difference in pain catastrophising levels captured in the lockdown, relative to baseline, period; t(84) = −0.54, p *=* .59. However, the mean self-perceived change in pain levels for the baseline group was 34.26 ± 33.26 (mean ± SD), indicating a significant self-perceived increase; t(82) = 36.76, p *<* .001 which was comparable to that seen in the full pain cohort; t(413) = 0.14, p *=* .82.

**Table 2.** Mean pain intensity (± SD) and pain catastrophising scores in 85 chronic pain patients for whom the baseline, pre-lockdown data were available.

	Pre-COVID	Lockdown
Pain intensity	7.55 ± 1.63	7.26 ± 1.37
PCS	27.89 ± 14.08	27.29 ± 12.48

PCS: Pain Catastrophizing Scale.

To further investigate the nature of self-reported changes in pain levels in the subgroup during lockdown, we analysed the relationship between self-reported pain intensity changes and baseline levels of pain catastrophising recorded prior to the COVID-19 pandemic using Pearson’s correlation analyses. Baseline PCS scores demonstrated a significant correlation with self-reported perception of change in pain levels relative to lockdown periods; r(83) = .33, p *=* .003. This suggests that, although direct patient’s reported pain ratings in lockdown may not deviate significantly from those recorded prior to the pandemic, their perception of pain increases in a manner that aligns to individual differences in pre-existing pain catastrophising. A comparison of demographic, pain and psychological well-being data for the baseline pain group and pain respondents without baseline can be seen in Supplemental material 1.

### Effect of lockdown on chronic pain patients relative to non-pain participants

**Figure 1.**
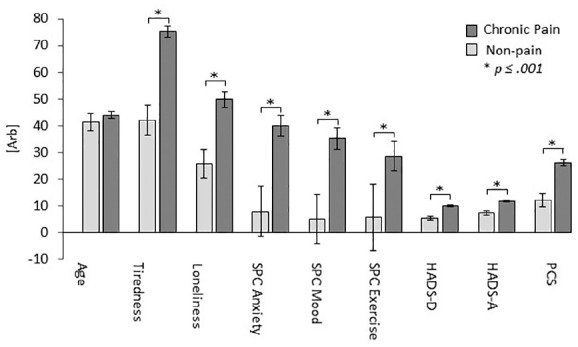
Mean self-reported levels of tiredness and loneliness, self-perceived lockdown-related increases in anxiety, depressed mood and reduction in exercise, HADS-A (anxiety) and HADS-D (depression) and pain catastrophising (PCS) scores in chronic pain and non-pain respondent groups with standard error bars.

We hypothesised that participants with chronic pain would demonstrate greater adverse effects of lockdown conditions, indexed by reporting of perceived increases in anxiety and depression, decreases in exercise (relative to the pre-lockdown periods) and increased scores for loneliness and tiredness. Independent samples t-tests (or Welch’s tests if the assumption of equality of variance was not met) were performed to compare mean ratings across all measures for chronic pain and non-pain groups. Bootstrapping (2000 samples) was used to estimate significance values while mitigating the likelihood of Type I error due to multiple tests. Results indicate that there were no differences between groups on demographics including age and the split of gender. For all variables, the chronic pain group reported significantly greater adverse effects, relative to non-pain participants. Figure 1 and Table 3 illustrate mean scores and comparison statistics for each group.

**Table 3.** Demographic parameters, self-reported levels of tiredness and loneliness, self-perceived changes (SPC) demonstrating lockdown-related increases in anxiety, depressed mood and reductions in exercise, HADS-A (anxiety) and HADS-D (depression) and pain catastrophising (PCS) scores in chronic pain and non-pain respondent groups.

	Chronic pain	Non-pain	t	df	p
Sex	90.7% Female	88.7% Female	−0.54	513	.55
Age	43.94 ± 13.01	41.21 ± 14.98	1.59	113.87	.10
Self-isolating	39.68%	6.81%	9.26	239.66	<.001
Any other illness	28.07%	6.81%	4.63	162.60	<.001
Anxiety SPC	39.36 ± 42.07	7.20 ± 42.24	6.53	517	<.001
Depression SPC	34.65 ± 41.39	4.11 ± 41.52	6.30	517	<.001
Exercise reduction SPC	28.69 ± 56.04	3.84 ± 57.39	3.77	512	.001
Tiredness	75.26 ± 21.03	41.66 ± 26.33	11.26	110.77	<.001
Loneliness	49.87 ±	25.43 ± 24.45	8.16	148.40	<.001
HADS-A	11.56 ± 4.42	7.37 ± 3.57	9.56	145.33	<.001
HADS-D	9.86 ± 4.34	5.37 ± 3.44	9.09	512	<.001
PCS	25.98 ± 12.76	12.26 ± 11.34	9.21	506	<.001

SPC: self-perceived changes; HADS: Hospital Anxiety and Depression Scale; PCS: Pain Catastrophizing Scale.

For each observed measure, means and standard deviations as well as group comparisons using t-test (or Welch’s test) are given with bootstrapped (2000 samples) significance values.

Chronic pain respondents self-reported greater lockdown-related increases in anxiety and depressed mood compared to non-pain group. They also report significant reductions in amount of exercise compared to pre-COVID period whereas negligible reduction was evident in the non-pain group. Chronic pain respondents scored higher on loneliness and tiredness ratings for past 7 days than the non-pain group. Unsurprisingly, increased HADS depression and anxiety scores and increased PCS scores were evident in the chronic pain, relative to non-pain respondents. Chronic pain respondents also reported increased levels of any other illness in prior 2 weeks (other than chronic pain), and they were more likely to be completely self-isolating due to high-risk status.

### Self-perceived changes in well-being and physical activity relate to self-perceived increases in pain during lockdown for chronic pain participants

We hypothesised that variance in levels of self-reported changes in psychological well-being and exercise would predict the degree of perceived increases in pain levels in our chronic pain population. Hierarchical multiple regression analysis was performed to investigate whether self-reported changes in anxiety, depressed mood and physical activity would predict levels of self-reported changes in pain intensity, after controlling for participant age, sex and reports of other illness in the past 2 weeks. Preliminary analyses were conducted to ensure no violation of the assumptions of normality, collinearity and homoscedasticity. In Step 1 of the model, the three confound variables were entered: participant age, sex and reports of other illness. This model was not statistically significant F (3, 415) = .43, p *=* .73 and explained 0.5% of variance in self-reported change in pain levels (Table 4). Following entry of self-reported changes in anxiety, depressed mood and exercise in Step 2, the total variance explained by the model was 11% (F (6, 415) = 8. 87; p *<* .001). The introduction of the predictor variables explained an additional 11% of variance in self-reported changes in pain, after controlling for participant age, sex and reports of other illness (R^2^ Change = .11; F (3, 415) = 16.96; p *<* .001). In the final adjusted model, two out of three predictor variables were statistically significant. Self-reported changes in exercise recorded the highest significance value (β = .17, p *<* .001) followed by changes in depressed mood (β = .17, p *=* .008). Changes in anxiety levels were a non-significant predictor (β = .11, p *=* .078).

**Table 4.** Hierarchical regression model of self-reported change in pain intensity.

	R	R^2^	R^2^ change	B	SE	β	t	p
Step 1	.071	.005	.005					
Sex				4.02	6.52	.03	0.62	.54
Age				0.08	0.14	.03	0.55	.58
Any other illness				4.35	3.99	.05	1.09	.28
Step 2	.34	.11	.11					
Sex				8.83	6.23	.07	1.42	.16
Age				0.13	0.13	.05	0.97	.33
Any other illness				1.46	3.81	.02	0.38	.70
Anxiety change				0.10	0.06	.11	1.77	.08
Mood change				0.15	0.06	.17	2.67	.008
Exercise change				0.11	0.03	.17	3.55	<.001

Step 1 describes the inclusion of confound variables prior to the analysis of predictor variables in Step 2. R^2^: variance explained by IVs; R^2^ change: additional variance in dependent variable; B: unstandardised coefficient; β: standardised coefficient; SE: standard error; t: estimated coefficient; p: significance value.

### Pain catastrophising relates to self-perceived increases in pain during lockdown and mediates the impact of depressed mood in chronic pain participants

In the subgroup of patients with baseline data, PCS scores from the period before the pandemic demonstrated a significant correlation with perceived levels of change in pain. To investigate whether pain catastrophising could act as a predictor, and/or mediator, of the relationship between self-perceived changes in mood and exercise and perceived levels of change in pain during lockdown, a mediation regression analysis was performed. The prior hierarchical multiple regression model was repeated with the addition of an intermediary step. After controlling for the confound variables, PCS scores were entered as a mediating variable, before the differential predictors were finally entered. As before, participant age, sex and reports of other illness were entered in Step 1 of the model which was not statistically significant F (3, 415) = .67, p *=* .55 (Table 5). Following entry of PCS scores in Step 2, the total variance explained by the model was 12% (F (4, 414) = 13.57; p *<* .001). PCS scores accounted for an additional 11% of variance in self-reported changes in pain, after controlling for participant demographics (R^2^ Change = .11; F (1, 411) = 51.96; p *<* .001). After the inclusion of self-reported changes in mood, anxiety and exercise levels in Step 3 of the model, the total variance explained was 18% (F (4, 411) = 12.63; p *<* .001). The introduction of the differential score predictor variables explained an additional 6% of variance in self-reported changes in pain, after controlling for confounds and PCS scores in the Step 2 (R^2^ Change = .06; F (3, 411) = 10.17; p = .001). In the final adjusted model, PCS scores exhibited the best predictive value (β = .27, p *<* .001) than self-reported changes in physical exercise (β = .15, p = .001). Change in mood was no longer significant (β = .11, p *=* .08), nor were perceived changes in anxiety levels t (β = .09, p *=* .17). The analysis indicates that PCS scores are also a significant predictor of self-perceived changes in pain levels during lockdown period. Pain catastrophising also acts as a partial mediator, as PCS scores accounted for the previously significant relationship between perceived changes in mood and pain, but not for predictive value of perceived changes in exercise.

**Table 5.** Hierarchical regression model of self-reported change in pain intensity including mediation via PCS scores.

	R	R^2^	R^2^ change	B	SE	β	t	p
Step 1	.071	.005	.005					
Sex				4.02	6.55	.03	0.61	.54
Age				0.08	0.14	.03	0.55	.58
Any other illness				4.35	4.01	.05	1.09	.28
Step 2	.34	.11	.11					
Sex				1.90	6.18	.01	0.31	.76
Age				0.22	0.13	.08	1.67	.10
Any other illness				0.59	3.82	.01	0.15	.88
PCS				0.98	0.14	.34	7.21	.00
Step 3	.42	.177	.06					
Sex				5.81	6.06	.04	0.96	.34
Age				0.23	0.13	.08	1.74	.08
Any other illness				−0.79	3.71	−.01	−0.21	.83
PCS				0.78	0.14	.27	5.63	<.001
Anxiety change				0.07	0.05	.09	1.37	.17
Mood change				0.10	0.06	.11	1.77	.08
Exercise change				0.10	0.03	.16	3.45	.001

R^2^: amount of variance explained by IVs; R^2^ change: additional variance in dependent variable; B: unstandardised coefficient; β: standardised coefficient; SE: standard error; t: estimated coefficient; p: significance value; PCS: Pain Catastrophizing Scale.

To further evaluate the role of pain catastrophising in the relationship between self-perceived changes in mood and pain, mediation analysis was performed using PROCESS toolbox for SPSS (http://www.processmacro.org/).^35^ Age, sex and reports of any other illness were included in the analysis as covariates of no interest. Bootstrapping procedures with 5000 samples and confidence intervals (CIs) of 95% were employed.

**Figure 2.**
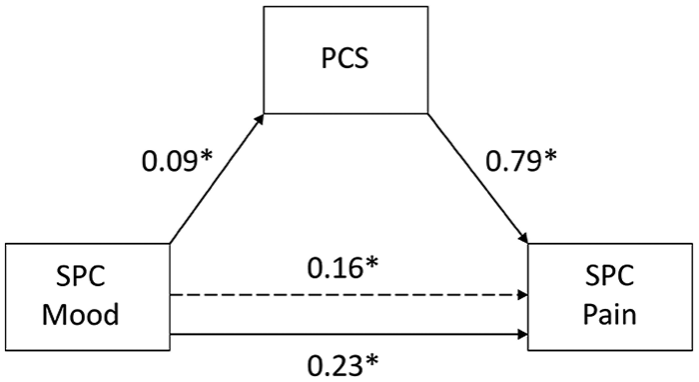
Relationships between self-perceived changes in mood and pain levels with pain catastrophising as mediator. Dotted line denotes the effect of perceived changes in mood on pain levels when the mediating variable of pain catastrophising is not included. All paths are reported as unstandardised ordinary least squares regression coefficients. SPC = self-perceived change; *p < .05.

After accounting for the effects of the covariates, self-perceived changes in mood were significantly related to changes in pain levels with, and without, the inclusion of pain catastrophising levels as a mediator (Figure 2). The analysis confirmed a significant mediating effect of individual levels of pain catastrophising on the relationship between self-perceived changes in mood and pain levels (indirect effect = 0.07, standard error (SE) = 0.02, 95% CI = 0.04 to 0.11).

## Discussion

We set out to understand how the COVID-19 pandemic, and associated lockdown restrictions, impacted individuals with chronic pain in terms of their psychological well-being, physical activity levels and pain experience. The findings reveal that people with chronic pain reported self-perceived increases in levels of pain severity during the most stringent period of lockdown in the United Kingdom (mid-April to early-May 2020) compared to the period before lockdown. They were more adversely affected by lockdown conditions than pain-free individuals, reporting greater self-perceived increases in anxiety and depressed mood, increased loneliness and reduced levels of physical exercise. People with chronic pain were more likely to be self-isolating due to high-risk status (observing increased levels of social distancing and restrictions on activity) and more likely to report any other illness in the preceding fortnight compared to non-pain counterparts. We hypothesised a mediating role for pain catastrophising on perceived changes in pain during lockdown and its mental and physical health consequences. The extent of self-perceived increases in pain symptoms in individuals with chronic pain was magnified by greater levels of pain catastrophising, which also mediated the impact of decreased mood on perception of pain. Perceived decreases in levels of physical exercise also independently related to perceptions of increased pain. Interestingly, actual changes in pain severity (relative to pre-lockdown reports of pain measured in a subgroup with baseline data) did not change significantly. Yet patients in this subgroup still reported self-perceived pain increases during lockdown, which were also predicted by baseline levels of pain catastrophising. Overall, the findings suggest that, during this period of crisis, pain catastrophising and physical activity levels are potentially important targets for pain management interventions.

Pain catastrophising and reduced levels of exercise are both essential components of the fear-avoidance model of chronic pain.^22,23,36^ In chronic pain populations, pain catastrophising contributes to hypervigilance and fear related to pain and results in lower levels of psychological resilience.^37^ People with high pain catastrophising scores have been shown to avoid strenuous exercise.^38^ Research evidence from chronic pain patients demonstrates that catastrophising predicts psychological distress,^39^ avoidance of daily living activities and increased levels of physical dysfunction.^40^ Physical inactivity promotes physical deconditioning, which then exacerbates pain during activity to cause greater aversion in a cycle of fear avoidance.^22,41^ In this manner, pain catastrophising promotes behavioural responses which lead to exacerbation of pain and other symptoms in chronic pain patients contributing to reduced quality of life.^36^

In the present research, pain catastrophising mediated the relationship between lockdown-related changes in perceived pain and depressed mood which reflects a recent study of older chronic pain patients.^42^ Pain catastrophising was previously shown to mediate the relationship between negative interpersonal events and pain-related affective symptoms^43^ and also moderated effects of exposure to missile attacks on pain and depressed mood in chronic pain patients in Israel.^44^ Together, these studies highlight the importance of catastrophising in chronic pain populations during the response to negative, high-stress situations. No other studies have yet analysed levels of pain catastrophising in the general population during the COVID-19 pandemic. However, health anxiety, which causes one to amplify perception of bodily sensations or changes as symptoms of being ill and which impacts on chronic pain experience,^45^ was recently shown to be exacerbated by the current pandemic,^46^ particularly in vulnerable populations.^47^ In this study, perceived changes in anxiety levels did not relate to lockdown pain increases. This aligns with previous research suggesting that pain catastrophising predicts post-operative pain levels independently of anxiety and/or depression^48^ and outperforms anxiety as a predictor of experimental and clinical pain intensity in non-clinical populations.^49^

It was recently highlighted that public health, social, clinical and psychological factors point to the likelihood of increased risk of pain and other symptoms in chronic pain populations during the COVID-19 pandemic.^4^ The present findings confirm this risk and demonstrate that people with chronic pain are more adversely affected by lockdown conditions compared to pain-free individuals. Perceived increases in pain severity and psychological distress offer empirical support to calls for rapid measures to provide appropriate care provision to chronic pain patients throughout this period.^4,13^ Cognitive behavioural therapy for chronic pain has greatest effectiveness when specifically targeting high catastrophising patients^50^ and can be successfully delivered using remote technology to reduce pain catastrophising.^51^ Remote technologies also have the potential to deliver pain physiology education, which are effective in alleviating pain catastrophising.^52,53^ Physical exercise interventions also offer a flexible and potentially effective approach.^54,55^ Meta-analyses of telemedicine approaches for pain management provision and exercise therapy in chronic pain patients indicate positive outcomes that are broadly comparable to usual care^56,57^ and highlight that telemedicine options may be a suitable substitute when usual care is not possible. Based on our data, we contend that remote pain management approaches to reduce pain catastrophising (particularly in high catastrophising patients) and promote physical activity should be considered for rapid implementation during the current crisis.

The findings from the baseline group indicated that, although self-reported levels of pain severity are perceived to increase during lockdown, actual levels of pain reported are comparable to data recorded before the pandemic. There are a number of reasons why this may be the case. First, it could indicate that self-perceived increases in pain severity are not due to actual increases in physical pain, but more a consequence of increased psychological distress. In this study, baseline pain catastrophising levels predicted the degree of self-perceived pain increase in the baseline subgroup. Previously, prospective studies have shown that baseline pain catastrophising predicts severity of post-operative pain.^58,59^ As pain catastrophising was also the strongest predictor of self-perceived increases in pain in the full chronic pain cohort, this points to the need to make this a principal clinical outcome and target for telemedicine pain management. On the contrary, it must be noted that the baseline sample was selected from ongoing or previous research which utilised local pain clinics for recruitment. These respondents did exhibit some demographic differences (older, greater proportion of males) and significantly higher levels of pain severity relative to chronic pain respondents recruited through other methods (Supplemental material 1). There could also be differences due to the data collection methods, with lockdown data collected using online tools compared to face-to-face clinics which could promote demand characteristics. Furthermore, actual changes in pain were measured using a different question and response scale compared to retrospective change ratings, pointing to the possibility that the latter method may be more sensitive to measuring changes in pain, albeit less quantifiable in terms of actual pain severity. Overall, we caution that the finding of no actual pain increases, compared to pre-lockdown data, in the baseline subgroup should be interpreted with restraint.

The present research has some limitations. First the chronic pain group were more adversely affected on *all* included measures of interest, although we could not practically include every clinically relevant measure available. For example, pain acceptance and perceived self-efficacy could be important factors not captured here. The relevant tools to capture these contributors typically include items that discuss quality of life in the context of pain (e.g. ‘I lead a full life even though I have chronic pain’) in the Chronic Pain Acceptance Questionnaire.^60^ There was a high risk that the validity of such items would be negatively impacted by the confounding effect of lockdown restrictions on lifestyle. The decision to focus on pain catastrophising in this study also reflects the fact that negative thought patterns have been shown to be more closely related to outcomes of perceived pain severity than positive factors such as pain acceptance.^61^ The recruitment also resulted in a much greater proportion of female respondents. This was entirely driven by increased levels of uptake among females, despite the fact that advertising always utilised locations open to any sex or gender. It is also important to note that online survey methodologies are subject to specific limitations such as the inability to validate accuracy of responses and levels of participant engagement. To maximise participant response engagement, free text responses were interspersed throughout the survey and validity of free text input was manually checked. On the same line, use of self-report for changing pain levels and participants own judgements of what constitutes a ‘typical’ pre-COVID week are subject to known psychological influences in chronic pain populations, specifically memory recall and aggregation biases.^62^ Despite the necessity imposed by lockdown, these factors should be considered as limitations of the present methodology. Additionally, no data was collected on participants’ own perceived adherence to the lockdown guidance which could represent an important factor for consideration. Finally, as a cross-sectional design, it is not possible to infer the causal nature of relationships between the many biopsychosocial factors which could be impacted during the current pandemic. All participants were subject to consistent lockdown conditions when responding to the survey, but there was variability in the time spent experiencing restrictions prior to providing their responses. In light of this, it is worth highlighting that this study reports on the first phase of data in a longitudinal design. Current respondents will continue to report on these measures regularly across coming months. Longitudinal data will permit more complex analyses to consider causality of the relationship between pain severity, pain cognition, psychological and physical well-being, and permit greater consideration of the influence of lockdown in a manner that is sensitive to temporal changes. It will also allow for consideration of long-term impacts of lockdown restrictions which could yet have unforeseen and far-reaching implications.

To conclude, the current findings are important because they represent the first empirical data to highlight increased suffering in people with chronic pain during lockdown. Specifically, people with chronic pain reported self-perceived increases in pain levels as well as increased adverse effects of lockdown compared to the non-pain comparison group. The findings support the urgent need for additional research concerning efforts to adapt remote clinical provision and to consider whether adverse effects of lockdown on vulnerable populations warrant consideration when generating guidance and implementing restrictions for specific groups. We highlight a potentially important role for pain catastrophising and reduced physical activity in the experience of people who live with chronic pain during lockdown conditions. This is significant because it points to possible clinical targets for therapeutic and behavioural interventions during the current, and future, crises. With additional research, it may be possible to rapidly adapt efforts to target remote pain management towards reducing levels of pain catastrophising, particularly in high catastrophising patients, and promoting physical activity as the pandemic continues. However, it is important to note that to truly establish whether such measures would be beneficial would require prospective research support, preferably within a randomised controlled trial design.

## Supplemental Material

sj-docx-1-bjp-10.1177_2049463720973703 – Supplemental material for Adverse effects of COVID-19-related lockdown on pain, physical activity and psychological well-being in people with chronic pain Click here for additional data file. Supplemental material, sj-docx-1-bjp-10.1177_2049463720973703 for Adverse effects of COVID-19-related lockdown on pain, physical activity and psychological well-being in people with chronic pain by Nicholas Fallon, Christopher Brown, Hannah Twiddy, Eleanor Brian, Bernhard Frank, Turo Nurmikko and Andrej Stancak in British Journal of Pain
