# Supplementary material for: Adverse effects of COVID-19-related lockdown on pain, physical activity and psychological well-being in people with chronic pain
Source: Br J Pain. 2020 Nov 21;15(3):357–68. doi: 10.1177/2049463720973703 (PMC8339954; doi:10.1177/2049463720973703)
Supplement: sj-docx-1-bjp-10.1177_2049463720973703 – Supplemental material for Adverse effects of COVID-19-related lockdown on pain, physical activity and psychological well-being in people with chronic pain [file sj-docx-1-bjp-10.1177_2049463720973703.docx]

**A1. Comparison of group with Baseline relative to chronic pain patients without baseline**

The sub-group (N=85) who enrolled in the research with baseline data available were recruited almost exclusively via contacts with a local tertiary care pain clinic. They demonstrated significant self-perceived increase in pain levels compared to the period before lockdown, but when their current pain and baseline intensity levels were compared there was no significant change evident. We speculated that the sub-group may have unique characteristics compared to the rest of the sample, which could impact reported pain levels at either time point. To test this, we compared demographic and self-report data between the subsample of patients and the rest of enrolled patients for whom no baseline data was available.. Independent samples t-tests (or Welch’s test in the case of violation of the assumption of homogeneity of variance) were performed to compare pain intensity scores in the lockdown period between the sub-group who had baseline data, and all other participants with chronic pain. The means and comparison statistics can be seen in Table A.1.

|  | **Baseline sub-group** | **All other chronic pain** | **t** | **df** | **P** |
| --- | --- | --- | --- | --- | --- |
| **Sex** | 80% Female | 93.5% Female | 3.2 | 98.11 | .002 |
| **Age** | 48.73 ± 13.21 | 42.55 ± 12.96 | 3.56 | 411 | <.001 |
| **Pain intensity** | 70.33 ± 15.02 | 65.39 ± 18.54 | 2.25 | 150.1 | .012 |
| **Pain SPC** | 34.61 ± 33.27 | 34.06 ± 36.8 | .14 | 413 | .82 |
| **Anxiety SPC** | 38.39 ± 40.17 | 40.12 ± 41.59 | -.31 | 413 | .76 |
| **Depression SPC** | 31.34 ± 45.2 | 35.55 ± 40.45 | -.82 | 413 | .46 |
| **Exercise SPC** | 35.97 ± 51.07 | 26.23 ± 57.7 | 1.36 | 413 | .15 |
| **Tiredness** | 76.62 ± 18.47 | 74.69 ± 21.8 | .74 | 411 | .46 |
| **Loneliness** | 48.49 ± 30.76 | 50.25 ± 30.55 | -.47 | 411 | .64 |
| **HADS-A** | 11.65 ± 4.2 | 11.52 ± 4.42 | .24 | 411 | .82 |
| **HADS-D** | 10.97 ± 4.65 | 9.56 ± 4.21 | 2.6 | 411 | .015 |
| **PCS** | 25.98 ± 12.76 | 12.26 ± 11.34 | 2.39 | 411 | .23 |
| **Pain interference** | 6.8 ± 2.04 | 6.13 ± 2.32 | 2.85 | 411 | .01 |

Table A1. Demographic parameters, self-reported levels of tiredness and loneliness, self-perceived changes (SPC) indicating lockdown-related increases in anxiety, depressed mood levels and reductions in exercise, HADS-A (anxiety) and HADS-D (depression) and pain catastrophizing (PCS) scores in chronic pain and non-pain respondent groups. For each observed measure, means and standard deviations as well as group comparisons using t-test (or Welch’s test) are given with bootstrapped (2000 samples) significance values.

The comparisons indicate that the sub-group were older, had a greater proportion of males and scored higher on levels of pain intensity, HADS depression, and BPI pain interference. Given these differences, it is feasible that the lack of actual changes in pain intensity reports in baseline compared to lockdown levels are due to increased levels of pain severity in this population (ceiling effect) and therefore this should be interpreted with caution.
